# Nonspecific Impact of Reflective Mind on Implicit Evaluative Processes: Effects of Experimental Manipulations and Selected Dispositional Factors

**DOI:** 10.3389/fpsyg.2017.01572

**Published:** 2017-09-11

**Authors:** Maria Jarymowicz, Anna Szuster

**Affiliations:** Faculty of Psychology, University of Warsaw Warsaw, Poland

**Keywords:** implicit affective priming, implicit self-reference effect, primary affect's independence effect, self-distinctness, evaluative heterogeneity, taking perspective of others

There is a growing interest in the phenomena of mind duality, one of the most intriguing properties of human nature. A review of classic texts (Maslow, 1954; Reykowski, 1975; Schneider and Shiffrin, 1977; Epstein, 1983) as well as more recent works (Gawronski and Bodenhausen, 2006; Clore and Huntsinger, 2007; Kahneman, 2011; Evans and Stanovich, 2013; Gawronski and Creighton, 2013; Sherman et al., 2014; Strack and Deutsch, 2014) shows that the authors refer to the distinctions between mental codes (affective vs. intellectual), cognitive and evaluative processes (associative vs. propositional), levels of consciousness, and regulatory systems (automatic vs. controlled). Advances in neurobiological research help to understand interrelations between diverse brain structures and regulative rules.

The alliance of psychology with neuroscience has become a standard in studies on emotions understood as evaluative processes (Ekman and Davidson, 1994; LeDoux, 1996, 2012; Oatley and Jenkins, 1996; Panksepp, 1998; Liberman, 2003; Sander and Scherer, 2009; Linquist et al., 2012; Imbir et al., 2015). The research conducted in our laboratory aims to differentiate between two evaluative systems: primary/automatic and reflective (Jarymowicz, 2008; Jarymowicz and Imbir, 2015). On the basis of the neurobiological and psychological models we assume that interactions between these two systems are reciprocal. The purpose of this opinion article is to present some empirical arguments related to the nonspecific impact of the reflective system on the automatic one.

## Basic characteristics of two evaluative systems: primary/automatic and reflective

The theoretical framework of our studies is based mainly on Zajonc's idea of the primacy of affect (Zajonc, 1980, 1984) and LeDoux's (1996) model of different roads of stimuli to the amygdala (“the emotional computer”; Sander et al., 2003)—the subcortical and the cortical ones. The research program attempts to define the specificity of the two diverse evaluative systems, their functions, and their reciprocal relationships (Jarymowicz, 2008, 2016; Jarymowicz and Imbir, 2015).

Our studies quoted below concern the primary automatic system generating evaluations based on subcortical processes (LeDoux, 1996, 2012) and implicit cognition leading to implicit appraisals (Sander and Scherer, 2009). Such affective reactions to stimuli occur in a mindless manner. All their attributes—origin, content, and effects (Gawronski et al., 2006)—can be totally implicit, that is introspectively unidentified (Greenwald and Banaji, 1995). Since primary affects are diffusive (Zajonc, 1980), they often influence explicit judgments concerning diverse objects, unrelated to unconscious stimuli (Murphy and Zajonc, 1993).

The reflective evaluative system, based on deliberate thinking, requires effort and is time- and energy-consuming. Moreover, evaluations can't be made without referring to cognitive, verbalized evaluative standards (Reykowski, 1989) which serve as mental bases for the object's appraisal in terms (often abstract ones) of good and evil. The evaluative processes are developed as a result of comparison between the actual, real circumstances and the conceptual, verbalized standards. Appraisals are made as a consequence of: (1) search for appraisals' premises, (2) piecemeal analyses of the object's properties, and (3) heterogeneous evaluations which, finally, reduce certainty and extremity of judgments (Jarymowicz, 2016).

Numerous data show a nonspecific influence of implicit affective stimuli on explicit judgments and behavior (e.g., Chen and Bargh, 1997; Berridge and Winkielman, 2003; Ohme, 2007). The explanations are based on the data showing that primary diffusive affects have an impact on subsequent explicit appraisals. Thus, verbalized judgments can be dominated by previous, even unconscious affective reactions to unrelated stimuli. Apparently this type of influence leads to numerous negative consequences. Hence, an important question arises: which processes may reduce the primary, nonspecific affective influence on thinking and judgments?

## The main assumption: the reflective system changes the rules of evaluative processes

Researchers are familiar with the spectacular case of Phineas Gage (Damasio, 1994), who, having suffered injury to the frontal lobes, lost the ability to control his own impulsive reactions. Today there is no doubt that the prefrontal brain modifies the processes evoked on the lower levels of the central nervous system (Gazzaniga, 2012). This implies, in psychological terms, that the impact of primary affective reactions (for instance, negative affect due to expression of subliminally exposed face) on judgments concerning unrelated objects (like Chinese signs) can be limited. According to our hypothesis, the reduction of primary affects' influence can be due not only to (1) a voluntary, reflective control over evaluative processes (when a subject is motivated to weight her/his own words), but also to (2) a nonspecific activation of the reflective system, which is connected with different rules of evaluation.

The results of gathered data show a specific influence of the subject's beliefs on her/his automatic reactions to implicit stimuli. This means that thinking and reasoning have an impact not only on the explicit knowledge and memory, but also on the implicit information processing (Uleman and Bargh, 1989; Underwood, 1996; Chen and Bargh, 1997; Holyoak and Morrison, 2005). However, can a nonspecific influence of the reflective evaluative system on the automatic evaluative system actually be evidenced?

There are reasons to posit that development of the reflective mind changes the rules of the entire mind's functioning. In particular, it can be assumed that the development of the reflective evaluative system leads to a habitual disposition to seek verbalized premises of one's own judgments. Also, its habitual character can result in relative independence from the influence of irrelevant affective processes on evaluative thinking. A series of our studies yielded some empirical evidence supporting these assumptions.

## Methodology: the affective implicit priming paradigms and measurements of their effects

The implicit priming paradigm constructed by Murphy and Zajonc (1993) was applied to all the studies mentioned below, although with some modifications. In the original version, participants were requested to intuitively evaluate unknown Chinese ideograms—allegedly “symbolizing human traits”—in terms of their negativity vs. positivity. Each sign was primed with a neutral or affective subliminal stimulus (exposed for 12 ms), i.e., a photograph of a face showing a neutral, positive, or negative expression. In some of our experiments, participants had to intuitively evaluate the extent to which a given ideogram “represents a trait” characteristic to the self—to measure the so-called *Implicit Self-Reference Effect* (Błaszczak and Imbir, 2012).

The original data analyses aimed at comparing the effects exerted by the type of experimental conditions (neutral × negative × positive priming) on the explicit evaluation of a neutral ideogram. This paradigm allows to show the impact of implicit affective priming on neutral stimuli evaluations (Ohme, 2007; Karwowska and Kobylińska, 2014).

An important modification of the affective priming impact index was introduced by Karwowska (2001), in order to differentiate between the participants who were more or less resistant to the influence of implicit affective stimuli. A difference between explicit appraisals made after exposure of negative or positive priming vs. the ones generated in control conditions (priming with a neutral stimulus) was calculated for each individual. The differences between appraisals following the affective vs. neutral priming show that explicit appraisals of a neutral stimulus (Chinese ideogram) are more or less neutral, and as such, relatively dependent upon/independent from the implicit affective priming.

## Nonspecific activation of the reflective system and registration of automatic reactions to implicit stimuli

In a series of studies, Karwowska (Karwowska and Kobylińska, 2014) applied the implicit affective priming paradigm. However, in the first stage of each study the author requested that participants present arguments concerning some social problems beforehand. For instance, they had to indicate positive and negative attributes of patriotism, or to enumerate arguments for and against acceleration of adoption procedures for children. The control groups dealt with a simple cognitive task: they had to compare pairs of numbers and say, in each case, if both were identical or different.

Next, the participants were invited to “a study on intuition.” They were asked to estimate the degree to which a trait (allegedly) symbolized by a given Chinese ideogram was negative or positive. Each ideogram was primed with suboptimal exposition of a photo of a face with neutral, negative, or positive expression. A repeated effect was found: the participants who were subject to a prior, deliberative thinking stimulation estimated neutral ideograms as significantly more neutral than the participants in the control conditions. We called this effect the “Primary Affect's Independence Effect” (PAI).

The PAI effect was measured in some studies aimed at identifying dispositional factors determining resistance to diffusive irrelevant affective influence on judgments and behavior (Jarymowicz, 2008). It was assumed that the relationships between automatic and reflective regulative systems are applicable not only to the conditions in which uncontrolled affective and controlled reflective processes are simultaneously stimulated. Further, it was presumed that reflective mind development leads to a kind of habitual readiness to seek evident evaluative judgment premises rather than guess what a given, unknown object or reality condition means. Based on the latter assumption, correlations between some selected dispositional factors and the PAI effect were predicted.

## Dispositional factors as possible correlates of resistance to the implicit affect influence on judgments

The studies' project was aimed to include some dispositional determinants of the PAI effect. As it was assumed that some dispositions have to be connected with reflective mind development, our interest fell on the determinants supposedly developed on the basis of reflective thinking. Three determinants had been chosen—all selected from our earlier personality research. (1) The first one—*the Self-Others Schemata Distinctness*–concerns the difference between prototypical traits ascribed (independently) by a subject to the self vs. to other people (Jarymowicz, 1987, 1991; Jarymowicz and Szuster, 2016). (2) The second one—*Evaluative Heterogeneity*—refers to the ability to perceive negative as well as positive attributes of the same object (Jarymowicz, 2016). (3) The third one—*Exocentric Altruism*—is related to centration on others and understanding the perspective of other people (Szuster, 2005; Szuster and Rutkowska, 2008).

All these variables are measured in a simple manner. The self-others schemata distinctness variable is measured by comparing the traits consecutively indicated by the subject as the most important for characterizing (a) other people and (b) the self. The evaluative heterogeneity variable is measured by the proportion of negative and positive attributes ascribed by the subject to a given object. The exocentric altruism is measured by way of manifesting the subject centration on other persons' states.

## Correlative studies: dispositional factors and reactions to the implicit affective stimuli

The hypotheses predicted that the degree of influence of implicit affective priming on estimation of Chinese ideograms will depend on the level of each of the dispositional variables. The same data pattern was found in all the studies (Jarymowicz, 2008): the higher the indices of self-distinctness, evaluative heterogeneity, or exocentric altruism, the more neutral the explicit estimations of the neutral Chinese ideograms (allegedly human traits) implicitly primed with photos of faces with negative or positive expressions.

In another study (Jarymowicz, 2008), self-distinctness and the *Implicit Self-Reference Effect* were measured. The result was consistent with the above-mentioned research: the higher the indices of self-distinctness, the lower the degree of reference of neutral, unknown Chinese ideograms primed with photos of unknown faces to the self.

Thus, all the gathered data indicate that self-others schemata distinctness, evaluative heterogeneity, and exocentric altruism—as measurements of dispositional variables—correlate positively with a kind of resistance to the influence of the implicit affective priming on one's own judgments.

## Conclusions

Numerous arguments allow to differentiate between the reflective evaluative system connected with specific evaluative rules and the system evoking automatic affective reactions. The empirical studies show that implicit affective stimuli can have a nonspecific impact on explicit judgments. On the other side, the data mentioned above suggest that resistance to implicit stimulation can be caused by the nonspecific impact of the reflective system on the automatic one. The results were similar in the two types of studies mentioned: those in which deliberative thinking stimulation was applied, and those in which some reflective dispositions were measured.

In more general terms, it can be assumed that reflective system development leads to changes in the system of principles controlling automatically evoked affects. In particular, the data suggest that the reflective evaluative system may inhibit influence of primary affective reactions on judgments.

## Author contributions

MJ: Substantial contributions to the general assumptions, data analyses, and interpretations; Draft and the final version of the Opinion Article (with the common approval of all parts of texts by both authors). AS: Contribution to the part of design of studies on the exocentric altruizm, data analyses, and interpretations; Draft and the final version of the Opinion Article (with the common approval of all parts of texts by both authors).

### Conflict of interest statement

The authors declare that the research was conducted in the absence of any commercial or financial relationships that could be construed as a potential conflict of interest.
